# The “Great Debate” at Melanoma Bridge 2020: December, 5th, 2020

**DOI:** 10.1186/s12967-021-02808-3

**Published:** 2021-04-07

**Authors:** Paolo A. Ascierto, Michael B. Atkins, Alexander M. Eggermont, Jeffrey E. Gershenwald, Jean-Jacques Grob, Omid Hamid, Vernon K. Sondak, Jeffrey A. Sosman, Hussein A. Tawbi, Jeffrey S. Weber, Corrado Caracò, Iman Osman, Igor Puzanov

**Affiliations:** 1 Department of Melanoma, Cancer Immunotherapy and Innovative Therapy, Istituto Nazionale Tumori IRCCS “Fondazione G. Pascale”, Naples, Italy; 2 Georgetown-Lombardi Comprehensive Cancer Center, Washington, DC USA; 3 Princess Maxima Center and University Medical Center Utrecht, Utrecht, The Netherlands; 4 Department of Surgical Oncology, The University of Texas MD Anderson Cancer Center, Houston, TX USA; 5 Service de Dermatologie et Cancérologie CutanéeHôpital de la Timone, Aix-Marseille Université, Marseille Cedex 5, France; 6 The Angeles Clinic and Research Institute, Los Angeles, CA USA; 7 Department of Cutaneous Oncology, Richard M. Schulze Family Foundation, Moffitt Cancer Center , Tampa, FL USA; 8 Departments of Oncologic Sciences and Surgery, University of South Florida Morsani School of Medicine, Tampa, FL USA; 9 Robert H Lurie Comprehensive Cancer Center of Northwestern University, Chicago, IL USA; 10 University of Texas MD Anderson Cancer Center, Houston, TX USA; 11 Laura and Isaac Perlmutter Cancer Center, NYU School of Medicine, New York, NY USA; 12 Unit of Melanoma and Skin Tumor Surgery, Istituto Nazionale Tumori IRCCS “Fondazione G. Pascale”, Naples, Italy; 13 New York University Langone Medical Center, New York, NY USA; 14 Department of Medicine, Roswell Park Comprehensive Cancer Center, Buffalo, NY USA

**Keywords:** Melanoma, Staging, Immunotherapy, Anti-PD-1, Anti-CTLA-4, Targeted therapy, BRAF inhibitor, MEK inhibitor, Adjuvant, Neoadjuvant

## Abstract

The Great Debate session at the 2020 Melanoma Bridge virtual congress (December 3rd–5th, Italy) featured counterpoint views from experts on five specific controversial issues in melanoma. The debates considered whether or not innate immunity is important in the response to cancer and immunotherapy, how useful are the revised American Joint Committee on Cancer (AJCC) classification for the staging of patients, the use of sentinel node biopsy for staging patients, the use of triplet combination of targeted therapy plus immunotherapy versus combined immunotherapy, and the respective benefits of neoadjuvant versus adjuvant therapy. As is usual with Bridge congresses, the debates were assigned by meeting Chairs and positions taken by experts during the debates may not have necessarily reflected their own personal opinion.

## Introduction

The Great Debate session at the 2020 Melanoma Bridge virtual congress (December 3rd–5th) featured counterpoint views from experts on five specific controversial issues in melanoma. The debates considered whether or not innate immunity is important, the revised American Joint Committee on Cancer (AJCC) classification for the staging of patients, the use of sentinel node biopsy for staging patients, triplet combination of targeted therapy plus immunotherapy versus combined immunotherapy, and neoadjuvant or adjuvant therapy. As with previous Bridge congresses, the debates were assigned by meeting Chairs and positions taken by experts during the debates may not have necessarily reflected their respective personal view. Audiences voted both before and after each debate.

## Is innate immunity important: yes or no?

### Jeffrey S. Weber: yes

Although there are few direct trials of innate immune effectors or drugs that impact them directly in melanoma, this is a new field that is being actively explored in other cancer types with promising results. Much of the impact of innate immunity is suppressive, i.e., through myeloid-derived suppressor cells (MDSCs), tumor-associated macrophages (TAMs), neutrophils and monocyte-secreted cytokines. Thus, evidence for the role of the innate immune system in melanoma and its effect on checkpoint inhibition therapy is indirect and is largely based on observational and depletion studies.

Several studies have investigated the prognostic impact of the neutrophil-to-lymphocyte ratio (NLR) in patients with melanoma. A meta-analysis reported that a higher NLR was associated with worse survival outcomes [1]. More recently, elevated baseline serum interleukin (IL)-8 was associated with poor outcomes in patients with advanced cancers treated with checkpoint blockade (nivolumab and/or ipilimumab) or chemotherapy [2]. In addition, a clear relationship has been shown between high levels of systemic and tumor-associated IL-8 and reduced clinical benefit from programmed death (PD)-1 blockade in patients with metastatic urothelial or renal cell carcinoma [3]. IL-8 was primarily expressed in circulating and intratumoral myeloid cells and high IL-8 expression was associated with downregulation of the antigen-presentation machinery, such as human leukocyte antigen (HLA) genes and interferon (IFN)-γ-induced genes. These data suggest IL-8 generated by innate immune cells has a negative suppressive role.

Baseline serum IL-6 levels are also associated with decreased overall survival (OS) in metastatic melanoma. Analyses of three immune checkpoint blockade trials showed worse survival in patients with higher baseline levels of IL-6, including patients receiving chemotherapy, indicating that IL-6 is both a prognostic as well as a predictive factor [4]. In a study of patients with stage IV melanoma, higher plasma concentrations of either IL-6 or IL-8, or both, were associated with worse survival [5]. Patients with low IL-6 and IL-8 also had decreased circulating MDSCs, and low levels of MDSCs were associated with better OS. Baseline innate MDSCs have also been associated with poor OS and overall response rate (ORR) to PD-1 blockade [6]. Thus, innate myeloid and neutrophil cells producing IL-6 and IL-8 are associated with a poor outcome with checkpoint blockade in melanoma, and both present a potential therapeutic target. Ergo, innate myeloid cells are associated with a poor outcome in melanoma and are important inhibitors of checkpoint blockade.

Gamma-delta T cells reside at the interface of innate and adaptive immunity. Gamma-delta 1 and 2T cells are the main subpopulations and account for up to 10% of circulating lymphocytes in healthy persons. Gamma-delta 2T cells recognize metabolites of host mevalonate and the microbial non-mevalonate pathway, while gamma-delta 1T cells recognize candidate antigens of stressed and tumor cells, an immune response associated with different pathogens. Gamma-delta T cells represent the major lymphocyte population infiltrating melanoma, with both gamma-delta 1 and 2 cells involved, suggesting that an innate immune response mediated by gamma-delta T lymphocytes may contribute to the immunosurveillance of melanoma [7].

Natural killer (NK) cells are innate lymphocytes with cytotoxic activity against cancer cells mediated by the release of cytokines and chemokines. They participate in immune responses against solid and haematopoietic cancers owing to their capacity to recognise characteristic molecular patterns of stressed cells and are able to recognize cancer cells without requiring neoantigen or self-antigen overexpression. Loss of major histocompatibility complex (MHC) expression increases the susceptibility of tumor cells to NK cell-mediated death. Killer cell immunoglobulin-like receptors (KIRs) are expressed on the surface of NK cells, and different KIRs recognise and bind different HLA ligands. NK cells are short-lived effectors within tumors that also express PD-1. An NK gene signature in a study assessing The Cancer Genome Atlas (TCGA) was associated with survival in melanoma, highlighting potential benefit associated with increased NK cell activity [8]. NK cells not only kill tumor cells but also recruit key immune cell populations required for protective tumor immunity via XC-chemokine ligand-1/2 [9]. NK cell effector cytokines such as XC-chemokine ligand-22 and FMS-related tyrosine kinase 3 ligand (FLT3LG) mediate dendritic cell (DC) recruitment and maintenance. Higher levels of circulating NK cells are also associated with improved OS in melanoma.

Allogeneic NK cells have been used in leukaemia, and chimeric antigen receptor (CAR) NK cells have shown promising results in lymphoma, with 8 of 11 patients with relapsed or refractory CD19-positive cancers having a response in one study [10]. In solid tumors, allogeneic NK cells have been investigated in combination with pembrolizumab in patients with previously treated advanced non-small-cell lung cancer (NSCLC). Pembrolizumab plus NK cell therapy resulted in improved survival versus pembrolizumab alone, with multiple NK cell infusions associated with better survival than a single infusion [11]

NKT cells are an unusual population of T cells recognizing lipids presented by CD1d, a non-classical class-I-like molecule, that are potent secretors of γ-IFN and that express IL-12 receptors and CD40L. In nine patients with minimal disease burden melanoma, adoptive transfer of invariant NKT cells was well tolerated and suggestive of potential anti-tumor activity [12].

HLA-E is expressed on many tumors, including melanoma, and high levels of soluble HLA-E are found in melanoma. Blocking HLA-E/NKG2A may overcome resistance of immuno-edited tumors. NKG2A is present on 50% of NK cells. Monalizumab is a humanized immunoglobulin G4 that blocks HLA-E/NKG2A interactions on NK cells. In PD-1 refractory microsatellite stable colon cancer, monalizumab in combination with durvalumab resulted in a 10% response rate [13], while durvalumab and monalizumab had a 20% response rate in PD-1 refractory head and neck squamous cell carcinoma (HNSCC) patients [13]. These data suggest NK cells are important in cancer, and may be so in melanoma.

In conclusion, innate immune cells are important for the activity of immunotherapy in melanoma, with neutrophils and myeloid cells associated with a poor outcome to immune checkpoint blockade. High IL-6 and IL-8 levels from myeloid cells and neutrophils are also associated with worse outcomes with immune checkpoint inhibition and strategies to overcome this by blocking IL-6 and IL-8 are being explored. Gamma-delta T cells have preclinical activity in murine melanoma and other tumor models and are being tested in melanoma. Both autologous and allogeneic NK cells, which are innate effectors, have activity in different cancers. Overall, the evidence suggests innate immunity is important for the success of immunotherapy and provides a mechanism for overcoming resistance to immune checkpoint blockade, in melanoma and other tumors.

### Alexander M. Eggermont: no

The immune system is blocked at multiple levels. However, the revolution in immunotherapy has been dominated by just two T-cell regulators, anti-cytotoxic T-lymphocyte-associated antigen (CTLA)-4, which protects and prolongs the priming of T cells, and anti-PD-1/PD-ligand (L)1, which activates T cells in peripheral tissues. Immunosuppressive components of the innate immune system, such as M2 TAMs and MDSCs, may also need to be targeted to improve outcomes.

Anti-FcγRII can be used to avoid anti-PD-1 transfer by macrophages, with anti-FcγRIIb resulting in continued CD8 effector activity. Alternatively, Fc modulation of immune checkpoint inhibitors may avoid anti-PD-1 transfer. Prolgolimab is an IgG1 PD-1 monoclonal antibody with a L234A-L235A (LALA) mutation that results in silencing of Fc binding and thereby avoids anti-PD-1 transfer [14, 15]. In the MIRACULUM trial of prolgolimab in patients with advanced melanoma, 2 year OS was 64.4% in 45 untreated patients with cutaneous melanoma [16, 17], which is comparable to that seen in the Checkmate-067 trial with combined nivolumab and ipilimumab.

The macrophage checkpoint is an anti-phagocytic interaction between signal regulatory protein alpha (SIRP-α) on macrophages and CD47 on all types of cells [18]. Antibodies against CD47 and SIRP-α are currently in development for a variety of cancers. Phagocytosis is maximized by inhibiting CD47 on ‘self’ cells or SIRP-α on macrophages in combination with antibodies that opsonize the target. Neither antibody blockade of CD47-SIRPα nor antibody opsonization of a target is sufficient to make target engulfment efficient, whereas the combination promotes phagocytosis.

M2-M1 repolarization agents include CCR5 and CCR5/CCR2, as well as IL-32. Treatment of murine melanoma with IL-32 improved dendritic cell function and triggered M1 polarization as well as CCL5 release in macrophages, resulting in CCR5-mediated CD8 + T cell infiltration into the tumor microenvironment and the eradication of cancer cells [19]. IL-32 reduced tumor growth and rendered immune checkpoint inhibitor-resistant tumors responsive to anti-PD-1 therapy without toxicity. IL-32 also correlates with response to anti-PD-1 treatment in patients with melanoma. Inhibition of CCR5 results in repolarisation of TAMs and anti-tumoral effects have also been shown in a phase I trial with a CCR5 antagonist in patients with liver metastases of advanced refractory colorectal cancer [20].

**Fig. 1:**
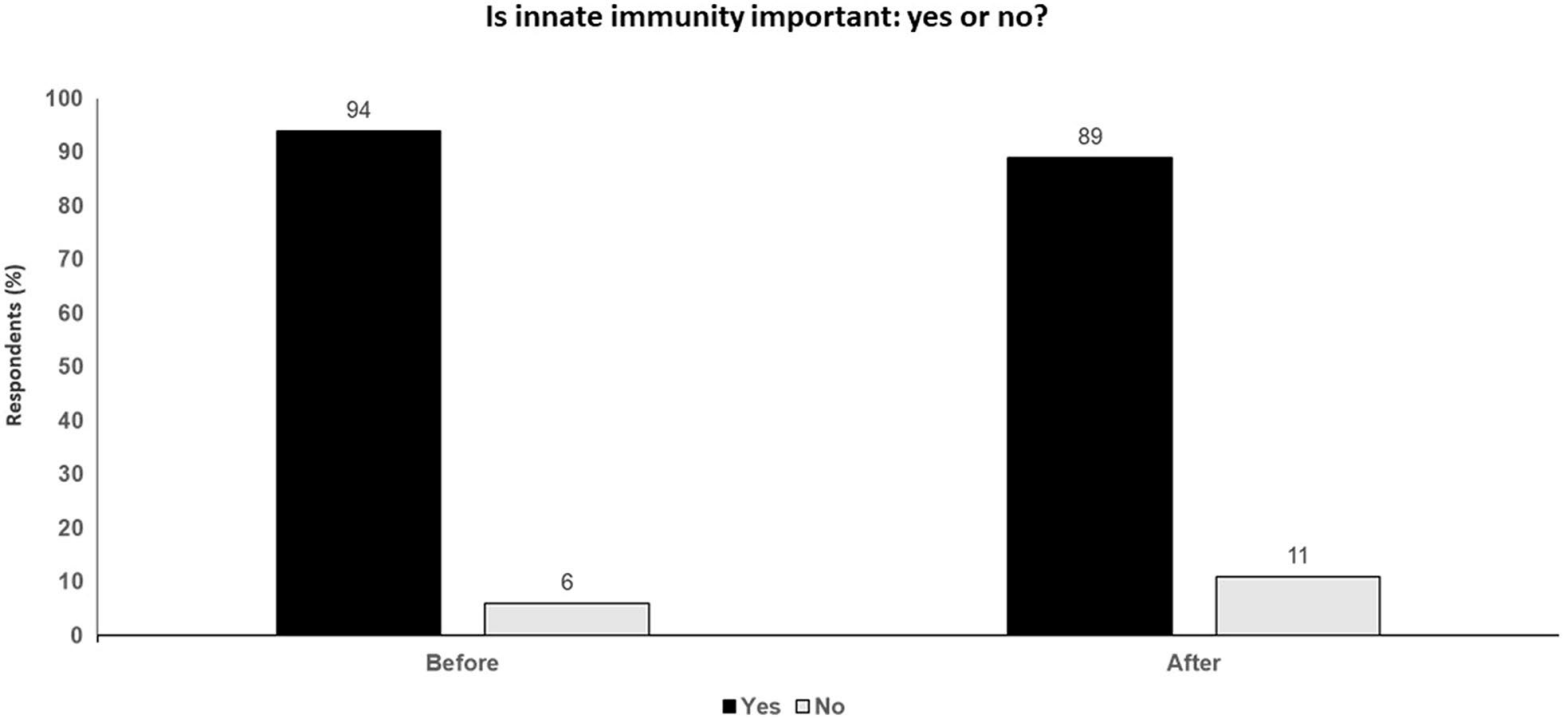
Is innate immunity important: yes or no? Audience response before and after debate

There are also various immune escape mechanisms, including loss of MHC Class I or B2M, loss of IFN- sensitivity (e.g., via IFN R1/2 mutation, JAK1/2 mutation), neoantigen depletion, immune-exclusion (e.g., WNT upregulation, PTEN loss, β catenin expression and upregulation additional checkpoint-inhibitors). However, these mechanisms are very complex and not easy to target individually and focusing on the macrophage population may be a more productive approach (Fig. 1).

### Key points

- The macrophage (mostly M2 macrophages)/MDSC component of the innate immune system is a profoundly immunosuppressive component of the tumor microenvironment in patients with cancer.
- Innate myeloid cells are associated with a poor outcome in melanoma and are important inhibitors of checkpoint blockade.
- Macrophage checkpoint inhibitors and M2-M1 repolarizing agents will become the next step in breaking tolerance and enhancing antitumor efficacy of a cytotoxic T lymphocyte-dominated immune response.

## Is the AJCC 8th melanoma staging system an improvement for better staging: yes or no?

### Jeffrey E. Gershenwald: yes

Melanoma staging provides the common language that helps to facilitate worldwide consistency in communication, our ability to speak to one another and to patients, and for registry reporting at multiple levels. Risk stratification from time of diagnosis, the latter a foundational element of the scope of the current staging system, defines patient groups by stage and prognosis and often informs treatment recommendations, clinical trial eligibility, and translational science.

The American Joint Committee on Cancer (AJCC) 8th edition (AJCC-8) attempts to reflect contemporary clinical practice. Key changes from the 7th edition (AJCC-7) include: for patients with clinically node-negative T2–T4 primary cutaneous melanoma, pathological nodal staging with sentinel lymph node biopsy (SLNB) was required for inclusion in survival analysis that informed revisinos, while T1 patients were included if SLNB biopsy was performed [21]. Tumor thickness is to be measured to the nearest 0.1 mm, and definitions of T1a and T1b were revised, recognising the importance of tumor thickness even within this group (T1a, < 0.8 mm without ulceration; T1b, 0.8–1.0 mm with or without ulceration or < 0.8 mm with ulceration). Tumor mitotic rate was removed as a T1 staging criterion, although it remains important and should be collected for all invasive melanomas, as prognostic models continue to be refined [22].

Compared with AJCC-7, AJCC-8 indicates a more favourable prognosis in stage I and II, largely because it includes a more homogeneous group of patients with stage I and II disease based on need for SLN assessment, when formerly many patients with thicker primary melanomas who had occult stage III disease were included, since SLNB was not required for patients to be included in the AJCC-7 analysis [23].

Patients with regional disease can have positive SLNBs, clinically detected regional nodes or non-nodal regional disease (in-transits, satellites and microsatellites). Non-nodal regional disease types were aggregated for staging purposes, based on similar survival outcomes in univariate analysis. N1a/b and N2a/b were unchanged, and N3 was expanded. The presence of microsatellites, satellites, or in-transit metastases are now categorized as N1c, N2c, or N3c based on the number of tumor-involved regional lymph nodes.

Prognostic stage III groupings based on N- and T-category criteria increased from three to four subgroups (stages IIIA–IIID) with recursive partitioning showing significant heterogeneity in disease-specific survival in a four substage group model. This change in the definition of stage groups has a significant impact on patient counselling, management and contemporary clinical trial design.

Concerns have been expressed over translating AJCC-7 to AJCC-8 [24]. However, the AJCC-8N and T category staging grid reflects biology of the disease, with thinner melanomas with 1–3 positive sentinel nodes having a more favourable prognosis (stage IIIA) while thicker melanomas that are more Iikely to be ulcerated and with more significant nodal burden are categorised as stage IIIC/D. This highlights the clinical impact of regional and primary tumor factors in driving stage III prognosis. Moreover, the ubiquity of electronic devices, as well as the evolving integration into electronic health records, means that these do not need to be memorised.

Compared with AJCC-7, AJCC-8 enables more accurate prognosis for patients with stage III melanoma. In a cohort of 1315 patients, AJCC-8 offered significantly enhanced prognostication for recurrence-free survival (RFS) [25].

Another benefit of the staging system is the ability to apply to recent clinical trials. In the KEYNOTE-054 trial of adjuvant pembrolizumab in high-risk stage III melanoma, subgroup analysis according to AJCC-7 and AJCC-8 staging at a median of 3-years follow-up showed robust prognostic separation was maintained [26]. Similarly, 5-year analysis of the COMBI-AD trial of adjuvant dabrafenib plus trametinib in patients with resected stage III melanoma also showed prognostic significance across strata, indicating that AJCC-7 data can be migrated across to AJCC-8 [27].

The AJCC-8 also embraces the importance of accurate SLN staging. Completion lymph node dissection (CLND) is no longer routine, and the additional ‘(sn)’ suffix is used to designate SLNB without CLND, adding granularity to the data.

For the M category, changes to distant metastases have been informed by contemporary clinical-decision-making, supporting clinical trial efforts in patients with advanced disease. In particular, there are enhanced definitions of the anatomic site with the expansion from three to four subcategories with the inclusion of M1d to designate the involvement of central nervous system disease; serum lactate dehydrogenase (LDH) is now stratified across all four M subcategories as not elevated or elevated.

Additional factors for clinical care are also recommended in AJCC-8, including primary tumor mitotic rate and SLN tumor burden. It is important to note that there are no single prognostic cut-off values for these factors. It is also clear that we are moving towards an era in which clinical decisions will be based on individualised prognostic risk assessment incorporating a multitude of factors, including clinical, pathological and, ultimately molecular and immune factors. Conventional staging will continue to inform but will not be the sole criterion. Biomarkers are important but are not yet sufficiently robust enough to be used in practice. A recent consensus on the use of gene expression profiling concluded that it had not been fully evaluated in the context of all clinical and pathological factors and that more evidence is needed to support informed recommendations on clinical care [28].

Looking ahead, AJCC cancer staging is likely to move to curated electronic system versions comprised of protocols. This will allow a more agile approach with iterative updates that can more effectively integrate clinically relevant advances. The use of non-anatomical factors will also be expanded. New arenas in melanoma will also likely be embraced, e.g., post-neoadjuvant treatment and its ‘yp/yc’ classification. The International Melanoma Database and Discovery Platform is an international collaboration to develop and validate integrated risk models and clinical tools that go beyond formal TMN staging.

In summary, AJCC-8 provides more homogeneous and better mapping to clinical decision-making in stages I–II while the integration of primary tumor and regional factors in stage III embraces the heterogeneity of prognosis to better inform the clinic. Moreover, the expansion and redefinition of M categories in stage IV better reflects contemporary understanding and clinical trial efforts. In addition, future considerations including the incorporation of non-anatomic factors and the development and implementation of integrated risk models will inform staging advances and the move towards online/electronic versions will embody a new more agile era.

### Jean-Jacques Grob: no

AJCC is based on the analysis of an epidemiological database with a cohort of over 49,000 stage I–II melanoma patients with long-term follow-up. However, this is not the only database, and we do not know whether it is truly representative of the real-world. Moreover, the ambitions of the AJCC classification are ambiguous and somewhat contradictory. It wants to be both a reference system to measure public health trends and to standardise the therapeutic benefit in clinical trials done, while also attempting to identify prognostic and adjuvant treatment predictive markers in melanoma. The first of these aims requires a stable tool that keeps constant variables and stages over time, while the latter involves being reactive and the inclusion of new markers.

Does AJCC-8 offer an improved reference system? Clearly no, since the main quality of reference standards is stability. The changes made to the system make it impossible to quantify the benefits of treatments over time, to treat patients according to the results of prior trials, or to understand epidemiological trends in melanoma. AJCC-8 was introduced at a time when stability was especially required because of major changes in practice, with no more CLND indicated after SLN-positive biopsy and the advent of new adjuvant treatments, and because some doubts were raised about the representativeness of the AJCC database. As CLND is no longer performed after positive SLNB, the status of nodes other than the sentinel node is no longer taken into account to stage the patients, and so a patient currently classified as AJCC-8 stage IIIA could have been considered IIIA, B or even C when node dissection was routinely performed. In addition, the applicability of trial results in the adjuvant setting is problematic since results were evaluated in AJCC7 and patients are now classified in AJCC 8th. The new AJCC-8 definition of IIIA disease has created a subset of patients with excellent prognosis, resulting in a debate about whether these patients should receive adjuvant treatment. This is a challenge for guidelines and healthcare agencies. Looking at the improvement in survival curves in stage IIIA patients between AJCC-7 and AJCC-8, it is impossible to know to what extent this is linked to changes in staging criteria, and what is due to improved management.

Does AJCC-8 offer any benefit in terms of prognostic markers? AJCC-8 has exactly the same limitations as all previous versions in that it is based on the same anatomical criteria which are virtually unchanged over decades. Potential new biomarkers (e.g., molecular, genetic, immune, microbiome) that could improve individual prediction are not included. Like AJCC7, AJCC8 does not predict better the outcome of a given patient. In AJCC 8 or 7th most melanoma deaths are issued from “low-risk” AJCC groups because they are much more numerous than “high risk” tumors. Better prediction would require markers that reflect the biological aggressiveness of a given tumor in a given patient at a given time. Unfortunately, AJCC-8 is no more reflective of biological aggressiveness than AJCC-7. Indeed, anatomical biomarkers (e.g., tumor thickness, SLNB) do not really represent the biological aggressiveness of the melanoma. For instance thick tumors may correspond either to an aggressive tumor, or to a non-aggressive tumor detected very late. Conversely, a thin tumor may correspond either to a non-aggressive tumor, but also to an aggressive tumor detected very early. Moreover, AJCC-8 is not even logical and intuitive as a prognostic staging, with many stage III (IIIA, B) patients having a better prognosis, than many stage II patients (IIC).

Is AJCC-8 superior to AJCC7 as a predictive marker? There is nothing new in AJCC-8 which could make it a better predictive marker for response to adjuvant therapy than AJCC-7. AJCC-7 substages (IIIA, B, C) did not influence the protective effect of adjuvant therapy. Hazard ratios (HR) were similar across all AJCC III substages in the KEYNOTE-054 and COMBI-AD trials. The same is observed when using AJCC-8 [26, Dummer 2020]. This means that the anatomical criteria of AJCC are not more relevant in the 8th than in the 7th version to predict response to adjuvant treatment with targeted or anti-PD-1 therapies. The only impact of the switch from AJCC7 to AJCC 8 in terms of predictive marker is to make it very difficult to confirm a benefit of adjuvant therapy in AJCC 8 IIIA patients. Indeed, the number of AJCC 7th IIIA patients enrolled in adjuvant trials who can now be restaged as IIIA AJCC8th is very low, and events are rare and occur late in this subgroup.

Changing AJCC will a be good idea if, and only if, one or several conditions are fulfilled: if new prognostic or predictive biomarkers are validated, if we no longer use sentinel node assessment, or if the database is changed to make AJCC more representative of real life.

**Fig. 2:**
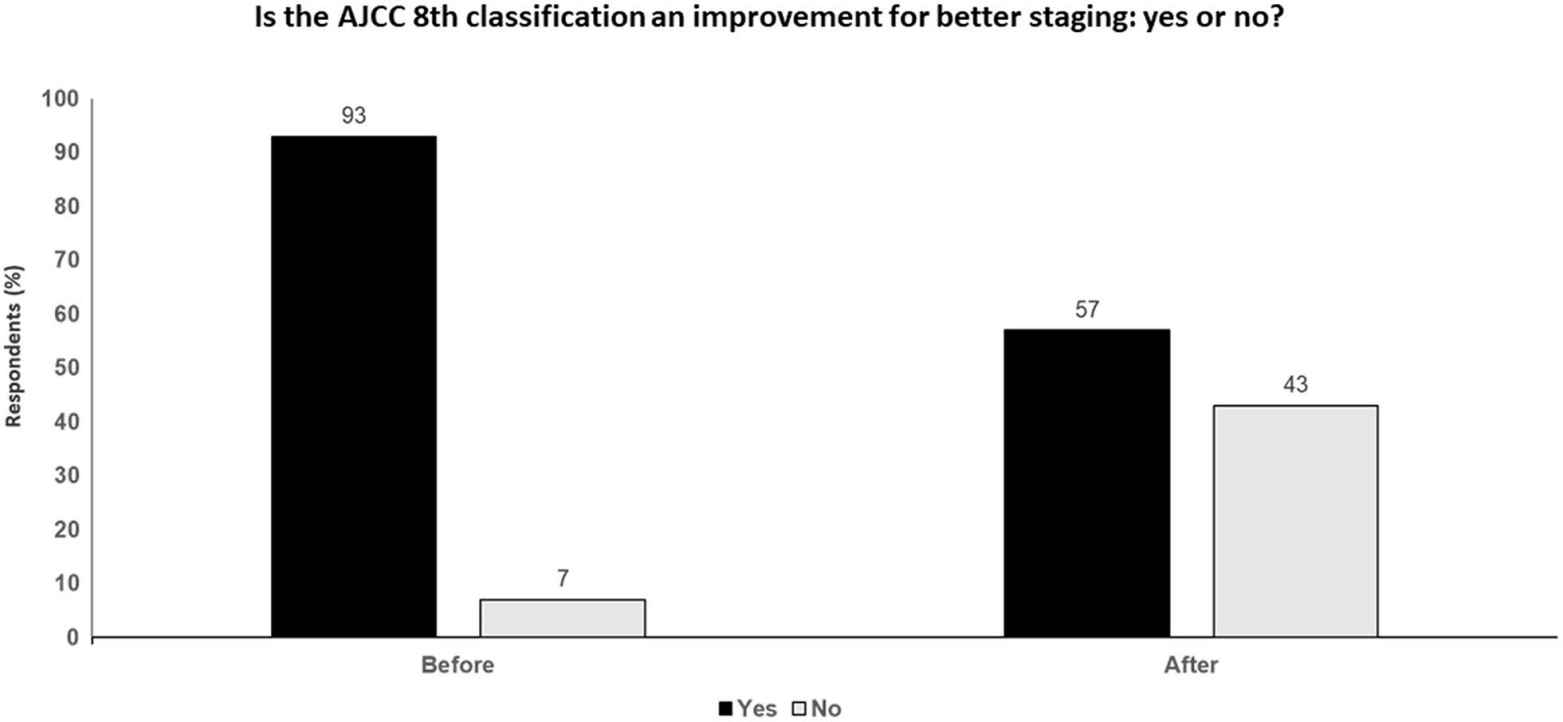
Is the AJCC 8th classification an improvement for better staging: yes or no? Audience response before and after debate

The only clear improvement with AJCC-8 is for stage IV patients with the introduction of major prognostic criteria, such as LDH and brain metastases. The other unexpected benefit is that AJCC-8 has indirectly shown that the sentinel node status may not be very useful. The fact that AJCC-8 IIIA patients do better than stage IIC, and similar to stage IIA and IIB patients, suggests the prognostic value of a positive sentinel node is not very important. AJCC-8 also provides a strong argument to promote adjuvant therapy in patients with stage IIA and IIB disease (Fig. 2).

### Key points

- Compared to AJCC-7, AJCC-8 provides more homogeneous and better mapping to clinical decision-making in stages I-II while the integration of primary tumor and regional factors in stage III embraces the heterogeneity of prognosis to better inform the clinic.
- However, new prognostic or predictive biomarkers need to be validated, and other databases which do not show the same absolute risk in the different stages need to be taken into account in order to make AJCC more representative of real life.
- AJCC-8 has indirectly shown that the sentinel node status may not be very useful as a prognostic factor, given that AJCC-8 IIIA patients do better than stage IIC, and similar to stage IIA and IIB patients.
- Future considerations including the incorporation of non-anatomic factors and the development and implementation of integrated risk models will inform staging advances and the move towards online/electronic versions will embody a new more agile era.

## Is sentinel node biopsy useful for staging patients: yes or no?

### Vernon K. Sondak: yes

Only 5 years ago, the surgical paradigm for clinically localised melanoma was wide excision and SLNB, including CLND for sentinel node-positive patients. Sentinel node-positive patents were preferably enrolled into a clinical trial, or treated with high-dose IFN or ipilimumab. Patients with recurrent disease were re-evaluated for surgery if possible and otherwise received systemic therapy. The overarching goal was to achieve the highest survival and the greatest degree of regional disease control. In comparison, in 2020, CLND is no longer performed in sentinel node-positive patents but instead active post-operative surveillance of lymph node basins is important. Patients are still ideally enrolled on a clinical trial but, if not, receive anti-PD-1 or BRAF/MEK targeted adjuvant therapy with dabrafenib plus trametinib. At the time of recurrence, patients are now evaluated for neoadjuvant systemic therapy before further surgery is considered. Thus, the goal has shifted slightly to achieving the highest survival with the fewest lymph node dissections while maintaining regional disease control.

In our center, SLNB is indicated for otherwise healthy patients with melanomas ≥ 0.8 mm thick in any anatomic site. Routine use of SLNB in this population provides reliable staging information with low morbidity and few nodal recurrences among sentinel node-negative patients, and may have therapeutic value for sentinel node-positive patients. Moreover, SLNB can decrease the need for lymphadenectomy. SLNB reliably predicts recurrence and death at 5 and 10 years for intermediate-thickness primary melanomas [29]. A node-positive patient identified by SLNB has a better 10-year outcome than a patient without SLNB who is allowed to recur. This is the potential therapeutic value of SLNB alone or with CLND in the sentinel node-positive patient. SLNB followed in all cases by CLND improves melanoma-specific survival (MSS) for node-positive patients with intermediate-thickness primary melanomas. However, how much if any of this improvement is due to the CLND is unclear. Radical node dissection is still indicated for patients with clinically detected positive nodes, with most patients undergoing postoperative adjuvant therapy and some undergoing postoperative radiation, and neoadjuvant systemic therapy increasingly being used for these patients. Whenever possible, needle biopsy and not tumor excision should be used to establish the diagnosis of stage III melanoma.

CLND is no longer routinely recommended for patients after a positive SLNB. ‘Low-risk’ SLN-positive patients do well without CLND, so active nodal surveillance is the appropriate approach for informed patients willing to comply with a careful surveillance regimen, and for all patients undergoing systemic adjuvant therapy. Nodal surveillance without adjuvant therapy is largely restricted to those with low-risk T1 or T2a primary tumors, a limited number of positive sentinel nodes (N1a or N2a) and limited tumor burden (maximum dimension < 0.2 mm). Between 0.2 and 2 mm tumor burden, patients are selectively either observed or adjuvant therapy is applied, particularly in the younger, healthier patients. Systemic adjuvant therapy is recommended in essentially all patients with > 2 mm tumor burden.

Morbidity is decreased by earlier lymph node dissection, so early detection of nodal recurrence is important [30]. CLND after a positive SLNB has lower morbidity than ‘watch and wait’ with lymph node dissection at recurrence. However, a lymph node dissection should not be done until and unless absolutely necessary, since no dissection has the lowest morbidity. All sentinel node-positive patients who do not undergo lymph node dissection should be carefully followed for regional recurrence. The appropriate surveillance regimen after a positive SLNB without CLND, with or without adjuvant therapy, typically involves regional nodal ultrasonography by an experienced team every 4 months for 3 years then every 6 months for 2 years, then annually for up to 10 years. Cross-sectional imaging of the chest, abdomen, pelvis and/or neck should be done at least annually, and ideally annual brain magnetic resonance imaging should also be performed. Localised nodal recurrence during adjuvant therapy should be treated by node dissection, not simply by changing systemic therapy.

Surgery is still important in the management of node-positive melanoma as part of a team approach, including the surgeon as part of the surveillance of the active nodal basin, but relies on systemic therapy to improve long-term outcomes.

### Jean-Jacques Grob: no

If we look at all the possible reasons that have been advocated in favour of SLNB, none is verified SLNB does not provide any information for a better surgical management. First, SLNB is of no interest to indicate radical nodal dissection after a positive SLNB, since, anyway, radical dissection after positive sentinel node has no significant effect on survival. Second, in the Multicenter Selective Lymphadenectomy Trial (MSLT) 1 trial, SLNB-positive driven wide excision with immediate lymphadenectomy had no impact on OS compared to systematic wide excision and postoperative observation of regional lymph nodes with lymphadenectomy if nodal relapse occurred [31]. Similarly, in MSLT-2, immediate SLNB-positive driven radical dissection had no significant impact on OS compared with delayed radical dissection only after relapse [29]. Thus, SLNB is useless either as an indication for nodal surgery or for the timing of nodal surgery.

SLNB is not either effective as a predictive marker for response to adjuvant therapy. In the KEYNOTE-054 trial, pembrolizumab demonstrated the same benefit across all stage III subgroups when using AJCC-7 or AJCC-8 [26, 32], suggesting that the relative protection is independent of the nodal load. The mortality risk can be higher in some SLNB-negative patients (AJCC-8 stage II) than some SLNB-positive patients (AJCC-8 stage III), with IIB and IIC patients having worse outcomes than stage IIIA patients. Anatomical biomarkers such as SLNB status are not good prognostic markers, since they may not represent the biological aggressiveness of the melanoma.

SLNB is not of a major interest as a prognostic marker. Although a negative SLNB may indicate a non-aggressive tumor not yet able to metastasize, it can also represent an early diagnosis of a highly aggressive tumor before it spreads to the node or even a tumor which metastasises directly to distant sites. These false negatives for biological aggressiveness (aggressive tumors despite negative SLNB) lead to exclusion of high risk patients from adjuvant therapy. Similarly, a positive SLNB may be a late diagnosis of a less aggressive tumor that has ultimately metastasised to the node. This false positives (non-aggressive tumors, despite positive SLNB) may lead to unnecessary exposure to adjuvant treatment. If SLNB was not performed anymore, some patients classified AJCC-IIIA would be classified I–IIA and would not receive adjuvant therapy. This would not represent a loss of chances, since these IIIA patients are anyway low risk and the absolute benefit of adjuvant treatment is weak in this group, whatever the relative protection offered. If SLNB was not performed anymore, patients currently stage IIIC with thick ulcerated tumors but nothing clinically or radiologically detectable in the nodes would be classified IIB–C and thus unfortunately miss adjuvant treatment, and have a loss of chances. However, they represent only a small proportion of those with IIIC disease and may, in the near future, be accepted for adjuvant therapy based on the results of ongoing clinical trials conducted in stage IIB and C.

SLNB has not benefited to the design of adjuvant trials in melanoma. The use of SLNB in AJCC has also had a deleterious effect on the design of the major adjuvant trials, which have excluded patients who may have benefited. Without SLNB as a standard of AJCC, adjuvant trials would probably have been conducted in a high-risk population defined as either T3A–B T4A–B or any clinically or ultrasound detectable nodes, which represents a more relevant population to risk than AJCC IIIA–C. Also, there would be no debate over whether or not AJCC-8 IIIA patients should receive adjuvant treatment due to their low risk, because this group would not exist.

SLNB does not improve the effect of adjuvant therapy. Ipilimumab plus nivolumab expands more and broader resident T-cell clones when given before (neoadjuvant) than after (adjuvant) node resection [33].

Finally, in real-life practice, SLNB is only useful as an administrative prerequisite to prescribing adjuvant therapy, since adjuvant trials have unfortunately been done in stage III patients. Hopefully, SLNB will no longer be required once adjuvant therapy shows efficacy in stage II disease or a better biomarker is validated and available.

**Fig. 3:**
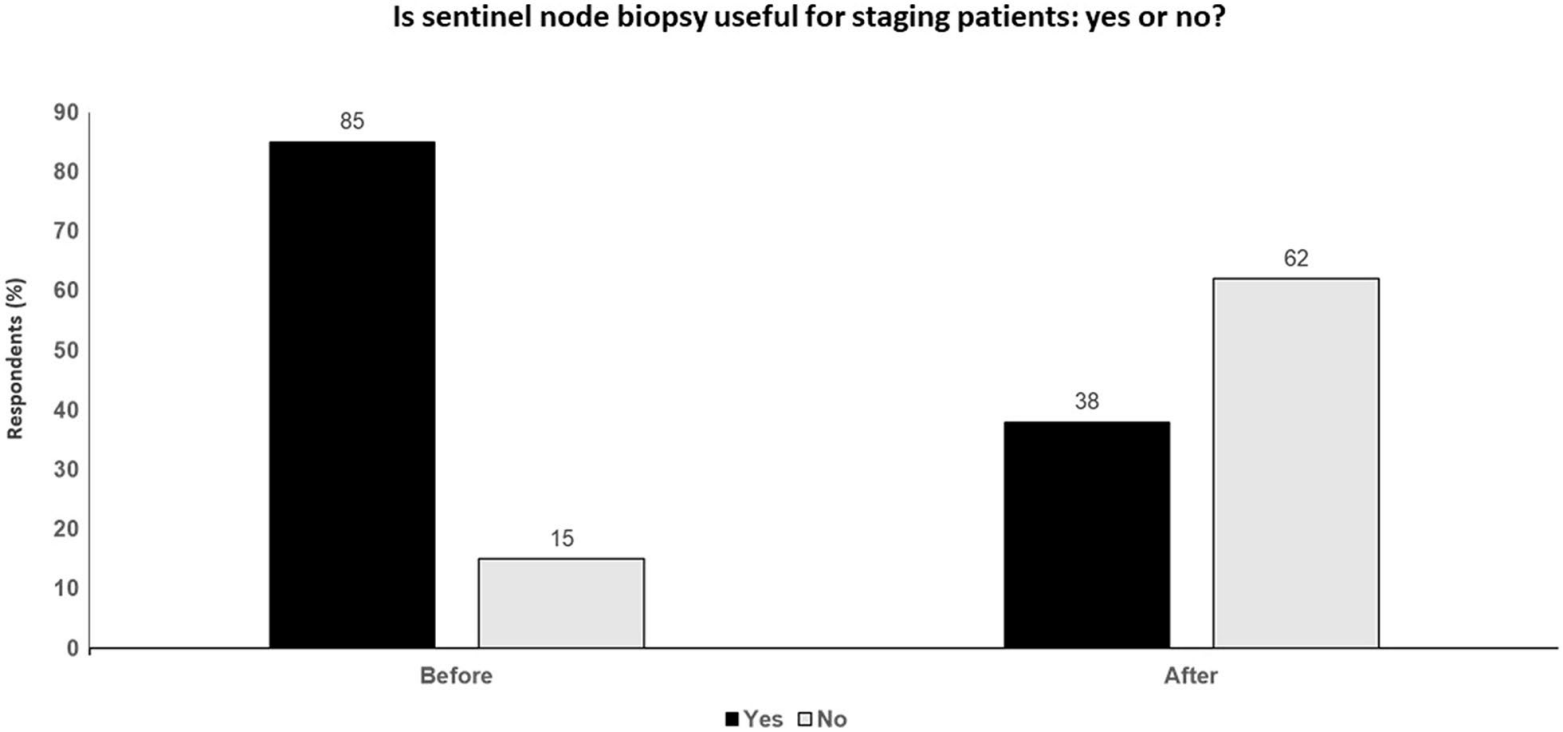
Is sentinel node biopsy useful for staging patients: yes or no? Audience response before and after debate

If the objective of staging is to improve clinical decision-making and patient outcome, then SLNB status is of very little interest, and we could simply rely on tumor assessment and node ultrasound until new biomarkers are validated (Fig. 3).

### Key points

- Routine use of SLNB in otherwise healthy patients with melanomas ≥ 0.8 mm thick in any anatomic site provides reliable staging information with low morbidity and few nodal recurrences among sentinel node-negative patients, and may have therapeutic value for sentinel node-positive patients.
- SLNB can also reduce the need for lymphadenectomy.
- ‘Low-risk’ SLN-positive patients do well without CLND, so active nodal surveillance is the appropriate approach for informed patients willing to comply with a careful surveillance regimen, and for all patients undergoing systemic adjuvant therapy.
- However, SLNB does not provide information that improves the outcomes after surgery, with radical nodal dissection after a positive SLNB having no significant effect on survival.
- SLNB will no longer be required once adjuvant therapy shows efficacy in stage II disease or a better biomarker is validated and available.
- SLNB status is of very little use in improving clinical decision-making and patient outcomes and tumor assessment and ultrasound are sufficient until new biomarkers are validated.

## Triplet combination (targeted therapy plus immuno-oncology therapy) versus combination immuno-oncology

### Jeffrey A. Sosman: in favour of the triplet combination

Preclinical data suggest combining an anti-PD-1 antibody with the BRAF inhibitor dabrafenib and the MEK inhibitor trametinib may enhance antitumor activity compared with dabrafenib plus trametinib alone [34]. Early phase I and II single arm trials suggested that anti-PD-1 plus BRAF inhibitor and MEK inhibitor may be associated with a higher percentage of patients with BRAF V600-mutant metastatic melanoma achieving more durable responses.

In the COMBI-d and COMBI-v randomized trials of targeted therapy, dabrafenib plus trametinib arms resulted in a 5-year progression-free survival (PFS) rate of 19% and a 5-year OS rate of 34% [35]. Elevated LDH was strongly associated with worse survival outcomes. In fact, those with normal LDH levels had even better 5-year PFS (25%) and OS (43%) rates. In the Checkmate-067 trial, combined immunotherapy with nivolumab plus ipilimumab resulted in 5-year PFS of 36% and 5-year OS of 52% [36]. Again, patients with normal LDH levels fared much better with regard to their OS.

Patients with BRAF mutation-positive melanoma have high ORRs with BRAF and MEK inhibitors. However, responses can be short-lived in many patients. Immune checkpoint inhibitors provide more durable responses, but response rates are generally lower. Preclinical and translational data demonstrate the immunologic effects of BRAF and MEK inhibitors, including influx of CD4 + and CD8 + T cells into tumors, decreased regulatory T cells (Tregs), decreased MDSCs, and upregulation of melanoma antigens [37]. Therefore, combining BRAF and MEK inhibitors with immune checkpoint inhibitors may overcome the clinical limitations of the individual classes of therapy and provide longer-lasting responses.

This hypothesis has been tested in three different trials. In the double-blind COMBI-I trial, dabrafenib and trametinib were combined with an anti-PD-1 antibody, spartalizumab and compared with dabrafenib and trametinib plus placebo in 532 patients with BRAF^V600^-mutated advanced melanoma. The addition of spartalizumab led to an improvement in the primary endpoint of PFS; however, this did not reach the primary endpoint with statistical significance (16.2 months in the triplet arm versus 12.0 months in the control doublet; HR: 0.820, p = 0.042) [38]. Although OS was not formally tested, a HR of 0.785 was observed in favor of dabrafenib and trametinib plus spartalizumab, although the median OS had not been reached in either treatment arm. Duration of response (DOR) also favored the triple combination.

In another trial, KEYNOTE-022, dabrafenib, trametinib and pembrolizumab resulted in an improvement over dabrafenib plus trametinib in the primary endpoint of PFS (16.0 versus 10.3 months in the doublet group, HR: 0.66; p = 0.043); however, the trial did not reach the planned benefit for a statistically significant improvement [39]. Median DOR was increased with triplet therapy (18.7 versus 12.5 months, respectively). No improvement in ORR was shown. In a later ad hoc analysis, the dabrafenib and trametinib plus pembrolizumab cohort showed superiority in PFS at 24 months (41% versus 16.3%), median duration of response (DOR) of 25.1 months versus 12 months, and OS at 24 months (63% versus 51.7%) in comparison to the BRAF plus MEK inhibitor arm.

The final trial reported that has investigated the benefit of the addition of an anti-PD-L1/PD-1 antibody to BRAF and MEK inhibitors in BRAFV^600^ mutant melanomas is IMSPIRE-150. In this trial, the addition of atezolizumab to vemurafenib and cobimetinib showed a statistically significant and clinically meaningful improvement in PFS versus vemurafenib and cobimetinib alone (15.1 vs 10.6 months; HR: 0.78; p = 0.025) [40]. At the time of analysis, OS data were not mature but favored the cohort receiving atezolizumab. ORR was similar in both cohorts. The addition of atezolizumab to vemurafenib and cobimetinib provided a clinically meaningful improvement in DOR versus vemurafenib and cobimetinib alone. The overall safety profile was consistent with the known risks of the anti-PD-L1 antibody and the vemurafenib and cobimetinib combination and no new safety concerns were identified.

These trials have shown that triplet combination therapy can increase PFS and DOR. Data on OS are too immature for any meaningful conclusion and there is no improvement in ORR. After progression on the BRAF plus MEK inhibitor arm, it is likely that all patients able to receive subsequent therapy will be treated with an anti-PD-1/PD-L1 antibody-based regimen certainly influencing the OS. One consideration is that these trials may not have selected the best control arm and perhaps anti-PD-1 would be a better comparator than targeted therapy. Another consideration that could be explored is the dosing schedule; alternate or sequential cycles or some other schedule may be more beneficial. Clearly, there is still a lot that needs to be learned to best integrate these two treatments but the underlying concept is of interest and worthy of further investigation.

## Michael B. Atkins: in favour of combination immuno-oncology (or sequenced immunotherapy and targeted therapy)

Ipilimumab is an important component of melanoma. For example, in Checkmate-067, combined immunotherapy with nivolumab plus ipilimumab resulted in an 8% improvement in 5-year OS versus nivolumab monotherapy (52% versus 44%) [36]. Moreover, 74% of patients treated with nivolumab plus ipilimumab who were still alive at 5 years were treatment-free, many for over 4 years. The impact on the addition of ipilimumab to nivolumab on survival is even greater in BRAF-mutant patients, with a 5-year OS rate of 60% versus 46% with nivolumab alone and 5-year PFS rate of 38% for nivolumab plus ipilimumab versus 22% for nivolumab monotherapy. Similarly, in the IMMUNED study in patients with resected stage IV melanoma with no evidence of disease, adjuvant nivolumab in combination with ipilimumab increased RFS compared with nivolumab alone [41], with this difference being even more pronounced in the BRAF-mutant population (2-year RFS of 87% versus 44% for nivolumab monotherapy, HR 0.17). Thus, ipilimumab clearly adds to the beneficial effect of anti-PD-1 therapy in patients with BRAF-mutant melanoma; however, it is not a part of current triplet combination regimens.

Treatment with BRAF inhibitors results in increased melanoma antigens and CD8 + T cells in tumors, which is associated with increased markers of T cell cytotoxicity, decreased immunosuppressive cytokines and vascular endothelial growth factor, and increased immunomodulatory molecules (PD-1, PD-L1 and TIM-3). This supports the hypothesis of potential synergy of BRAF-targeted therapy with immunotherapy, although important questions remain. For example, in murine melanoma, vemurafenib was associated with an increase in the number of CD3 + T cells in the tumor. However, this was also associated with decreased tumor volume and, when this is accounted for, vemurafenib did not increase the total number of immune cells in the tumor. In addition, in serial tumor biopsies in patients receiving BRAF ± MEK inhibitor therapy a comparable increase in CD8 + tumor-infiltrating lymphocytes (TILs) and other T cell subsets (e.g., CD3 + , FOXP3 +) is observed over time indicating that the observed T cell enrichment is not selective. As a consequence, there is no change in the CD4:CD8 ratio over time [42]. Additional studies did not reveal either a consistent TCRVB clonal expansion or significant increase in the proportion of antigen experienced or activated T cells as measured by IFN-γ or granzyme B expression. These data suggest that the perceived T cell influx seen with BRAF inhibitor therapy is not related to any enrichment of antitumor immunity, but instead is largely a consequence of relative depletion of tumor cells. Thus, the potential for synergistic activation of the immune system proposed as a rationale for triplet therapy might be the result of an artifact and thus illusory.

Three triplet combination studies have been reported, only one of which (IMspire150) [40] has met its primary endpoint of median PFS [38–40]. PFS at 12 months in the three studies is essentially equivalent across the triplet arms as well as the doublet arms. Response rates are also similar for the doublet and triplet combinations in each trial, and, in KEYNOTE-022, the ORR was actually higher with the dabrafenib plus trametinib doublet without the addition of pembrolizumab. When compared, the PFS rate of 38% at 5 years achieved in the patients with BRAF-mutant disease with nivolumab plus ipilimumab from the Checkmate-067 trial looks superior to that seen in any of the triplet arms in these three studies at 2 years.

Greater toxicity with the triplet combination relative to the BRAF plus MEK inhibitor doublets may also be a concern. In the IMspire150 trial, the reporting of toxicity was misleading in that approximately 25 patients in the triplet arm who could not tolerate the vemurafenib plus cobimetinib run-in period were switched to the placebo arm for purposes of safety reporting; these patients who did not tolerate the doublet likely would not have tolerated the triplet combination. The COMBI-i trial may provide a better indication of toxicity, with treatment-related serious adverse events and adverse events leading to treatment discontinuation (of at least one or all three study drugs) both higher in the triplet arm.

It is also difficult to identify which patients might be most appropriate for the triplet combination, as opposed to the BRAF plus MEK inhibitor doublet. Although it has been suggested that patients with the most aggressive disease might be most suitable for the triplet, data from IMspire150 do not support this view, with no PFS benefit seen with the triplet combination in patients with ECOG performance status 1, at least three organs involved, sum of longest diameters of target lesions (SLD) ≥ 44 mm, or M1c disease [40].

**Fig. 4:**
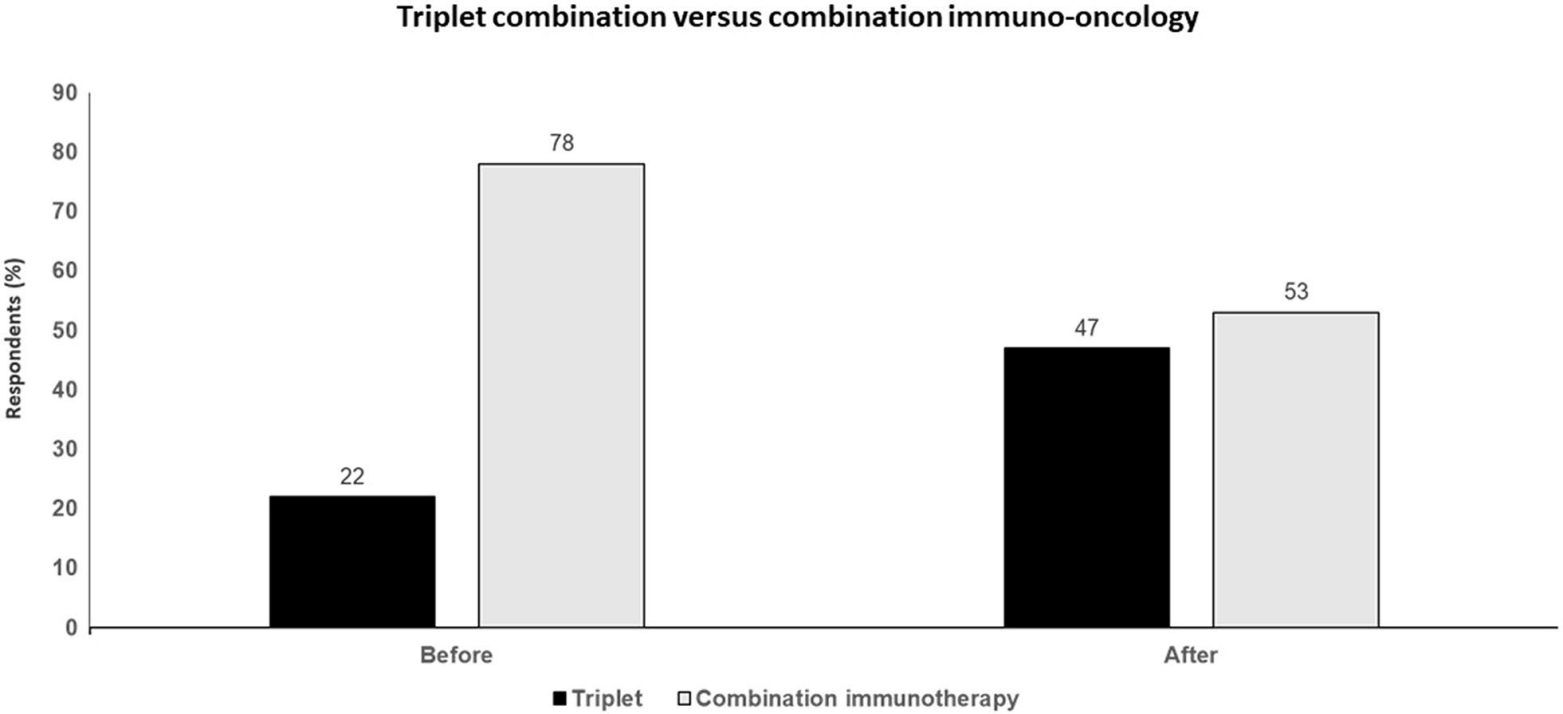
Triplet combination (targeted therapy plus immune-oncology therapy) versus combination immuno-oncology. Audience response before and after debate

Overall, the evidence suggests that the addition of PD-1 inhibitors to BRAF and MEK inhibitors results in slight improvements in median PFS, PFS at 1-year and DOR but no improvement in ORR. Moreover, the PFS plateau was not clearly evident but looks to be lower than the 5-year PFS rate of 38% with nivolumab plus ipilimumab. Full OS data are not yet available but it appears as though the plateau on the OS will be less than 50%, well below the 5-year OS plateau of 60% seen in patients with BRAF-mutant melanoma treated with nivolumab plus ipilimumab in the Checkmate 067 study. There is also no evidence for treatment-free survival, i.e., the ability to continue a tumor response in the absence of therapy, as was seen in 74% of patients surviving 5 years with nivolumab plus ipilimumab. Triplet combination therapy is associated with extra clinical toxicity and the addition of an extra drug will likely also involve added financial toxicity. Studies of the triple combination should have included nivolumab plus ipilimumab as the control arm rather than targeted therapy, since the results of such studies may have answered which regimen was better and so avoided this debate. Ultimately, it is difficult to identify a patient population that should receive triplet combination therapy rather than sequenced immunotherapy and targeted therapy (Fig. 4).

### Key points

- Triplet combination therapy can increase PFS and DOR but there is still a lot that needs to be learned to best integrate these treatments.
- The addition of ipilimumab to nivolumab is a particularly important component of the immunotherapy of patients with BRAF-mutant melanoma and is omitted from the current triplet regimens.
- Preclinical and clinical data suggesting immune activating effects of BRAF/MEK inhibitors may be more a consequence of tumor cell loss than novel T cell influx and portended the lack of synergy seen in triplet studies relative to BRAF/MEK inhibitors alone.
- Combination nivolumab plus ipilimumab results in higher PFS and OS rates at 5 years in patients with BRAF-mutant melanoma (with the majority of these being maintained in the absence of treatment) than are likely to be seen at 3 years with the triplets.
- Based on the triplet study subset analyses, it is hard to identify a patient population that benefits more from triplet therapy than sequenced immunotherapy and targeted therapy doublets.

## Neoadjuvant or adjuvant therapy: which is better?

### Hussein A. Tawbi: in favour of neoadjuvant

AJCC-8 has highlighted the significant decrease in MSS from stage I to stage III disease [21]. For patients with lymph node involvement, once clinically detectable disease is present there is a higher-risk of mortality. Patients with stage IIIC or D disease have a high rate of mortality.

Adjuvant therapy has clearly led to better survival in high-risk melanoma and is the current standard of care. Several studies, including COMBI-AD, Checkmate-238 and KEYNOTE-054, have shown significant improvements in RFS and a 50% increase in survival with immunotherapy or targeted therapy [26, 27, 43]. Longer-term, the proportion of patients expected to remain relapse-free with dabrafenib and trametinib in a cure rate model based on COMBI-AD was increased by 17% [44].

However, there are several issues with adjuvant therapy. A problem with adjuvant studies is that they are already skewed, given there is a > 15% screening failure rate prior to the start of adjuvant therapy due to relapse [45]. Adjuvant therapy can involve a high rate of relapse, with recurrence in over 40% of patients. In addition, over two-thirds of patients receive treatment without any benefit so are over-treated. There is also a lack of evidence to help guide clinicians in risk:benefit analysis during therapy. Finally, adjuvant studies require large numbers of patients and many years of follow-up.

A neoadjuvant approach provides the opportunity for systemic therapy ahead of surgery, with the potential for more aggressive treatment, e.g., with combination regimens. Treating existing disease is measurable and clinically and radiographically evaluable and pathological response can be evaluated. Toxicity can also be assessed in a short period. These assessments could help guide subsequent adjuvant therapy. Neoadjuvant therapy can also reduce the disease burden and can provide high quality and quantity of biospecimens for biomarker development and assessment.

Neoadjuvant therapy is a very active area of investigation with a large number of trials published and ongoing. However, these studies have been characterised by small patient numbers and a large degree of variability. The International Neoadjuvant Melanoma Consortium (INMC) was established in 2016 to harmonise clinical trial designs, align translational plans and efforts to understand biology of response and resistance, and to establish a platform for rapid drug development.

Despite their small size, trials to date have provided valuable insights into the role of neoadjuvant therapy. In an INMC pooled analysis of six neoadjuvant trials, immunotherapy and targeted therapy were associated with high pathological complete response (pCR) rates in patients with stage III melanoma and patients who achieved pCR, especially those on targeted therapy, were more likely to be recurrence-free at 12 months [46].

Compared with adjuvant therapy, neoadjuvant immunotherapy may result in the activation of many different T cells, with the activation, proliferation, and trafficking of tumor-specific T cell clones already within the tumor microenvironment. In the OpACIN trial, neoadjuvant ipilimumab plus nivolumab induced a high pathological response rate (78%), with all responders relapse-free at 3-year follow-up [33]. Moreover, neoadjuvant ipilimumab plus nivolumab expanded more tumor-resident T cell clones than adjuvant application. However, toxicity was high with 90% grade 3/4 toxicities, making the standard dose unfeasible for broader testing.

In the OpACIN-neo trial, IFN-γ and mutational load were associated with pathological response and relapse, with a 100% pathological response rate in patients with high IFN-γ and high tumor mutational burden [47]. B cell signatures were also enriched in the tumors of patients who responded to treatment. B cells and tertiary lymphoid structures contribute to immune checkpoint blockade response. Further analysis has indicated that tertiary lymphoid structures have an important role in the immune microenvironment in melanoma, by conferring distinct T cell phenotypes [48]. B-cell-rich tertiary lymphoid structures are also associated with survival and immunotherapy response in sarcoma [49].

Neoadjuvant therapy for high-risk melanoma has shown a correlation between pathological response and clinical outcomes, although this requires validation in prospective, randomized studies. It also opens a unique window into mechanisms of response and resistance, lending insights into more advanced melanoma and other diseases. The neoadjuvant setting also offers the opportunity to prioritise drugs for further development.

### Omid Hamid: in favour of adjuvant

Adjuvant therapy in melanoma is the standard of care based on many years of clinical trial evidence. Clinical trials have shown that ipilimumab, nivolumab, dabrafenib plus trametinib and pembrolizumab all provide a RFS benefit in patients at high-risk of relapse [32, 50–52]. Adjuvant trials have included large numbers of patients and data with long-term outcomes are available. For example, in the Checkmate-238 trial, 4-year RFS was 51.7% with nivolumab versus 41.2% with ipilimumab (HR 0.71; p = 0·0003) [43]. In contrast, neoadjuvant data are from studies with small numbers of patients and conclusions are largely based on the importance placed on pCR. There is an absence of randomized trials and insufficient evidence to make recommendations on the use of neoadjuvant therapy.

Data has shown that 97% of patients with a response to neoadjuvant therapy do not relapse within 24 months, compared to 52% among patients without response [46]. However, these are perfectly chosen patients in a non-randomized trial. Moreover, neoadjuvant trials to date have also involved adjuvant therapy for up to 1 year after surgery so it is not possible to separate their relative effects.

Subgroup analyses helps us understand which is the best therapy for our patients, but this is not available for neoadjuvant therapy. So, what are the available and mature data in support of a neoadjuvant approach? Data pooled from six trials included 211 patients [46]. Nineteen were excluded because of stage IV disease or in-transit metastases and a further 8 because of progressive disease or other reasons, leaving 184 patients with stage III nodal disease who underwent surgery. Five percent of patients will lose the opportunity to have potentially curative surgery if they receive neoadjuvant therapy. For targeted therapy, the RFS for adjuvant is clearly superior to neoadjuvant. For example, the 45% 2-year RFS rate achieved with neoadjuvant therapy is inferior to the 67% rate with adjuvant treatment in COMBI-AD [52]. RFS in stage IIIB and IIIC patients only was 60%, so still superior to neoadjuvant.

Providing neoadjuvant immunotherapy instead of facilitating secondary surgery could delay or complicate the procedure. Indeed, anti-PD-1 and anti-CTLA-4 agents are not known for their rapid antitumor effect, which could facilitate surgery. Rather, both checkpoint inhibitors might exert antitumor action in a delayed fashion and, in some cases, could result in a transitory tumor swelling, for which no validated imaging or biological method can reliably differentiate between an immune-mediated tumor flare and real tumor progression, or in the appearance of new metastases.

The neoadjuvant immunotherapy data at 12 months are impressive, with an RFS of 83% [46], compared with 64% in CheckMate 238 [51]. However, a better comparator is the IMMUNED study of adjuvant nivolumab plus ipilimumab or nivolumab monotherapy in patients with resected stage IV melanoma with no evidence of disease, which showed a 75% RFS at 1 year [41]. However, if these were stage IIIB-C resected patients, an increase of 5–7% might be expected, and if we take into account the 5% immunotherapy dropout rate from progression before surgery, the RFS rate with adjuvant therapy is close to the neoadjuvant figure.

The toxicity of the neoadjuvant treatments is also an important consideration. In particular, combination regimens have a high rate of adverse events which could delay surgery. Myths of the benefits of neoadjuvant therapy include that tumor shrinkage leads to decreased surgical morbidity. Although this might be true, such patients would be truly unresectable in the adjuvant setting so would instead receive systemic therapy. In addition, the idea that neoadjuvant therapy can result in an early immune response to multiple neoantigens, leading to the destruction of micrometastases and thereby prevent distant disease spread may have some validity. However, the same can be achieved with adjuvant therapy and there are many ongoing trials investigating ways to enhance neoantigen-specific T cell reactivity in the adjuvant setting, e.g., through the use of neoantigen vaccines. It is also easier to shift to the adjuvant setting from a metastatic one. One example of this is bempegaldesleukin in combination with nivolumab, which is being tested in an adjuvant trial following encouraging results in metastatic disease [53]. The belief that neoadjuvant therapy can provide an objective measure of a patient’s response to therapy and so lead to personalization of adjuvant therapy seems improbable, given what little has been achieved in this respect with metastatic and adjuvant therapy. The opportunity provided by neoadjuvant therapy to collect high-quality serial biospecimens to facilitate understanding of drug response and resistance is true but this is experimental and not standard practice.

**Fig. 5:**
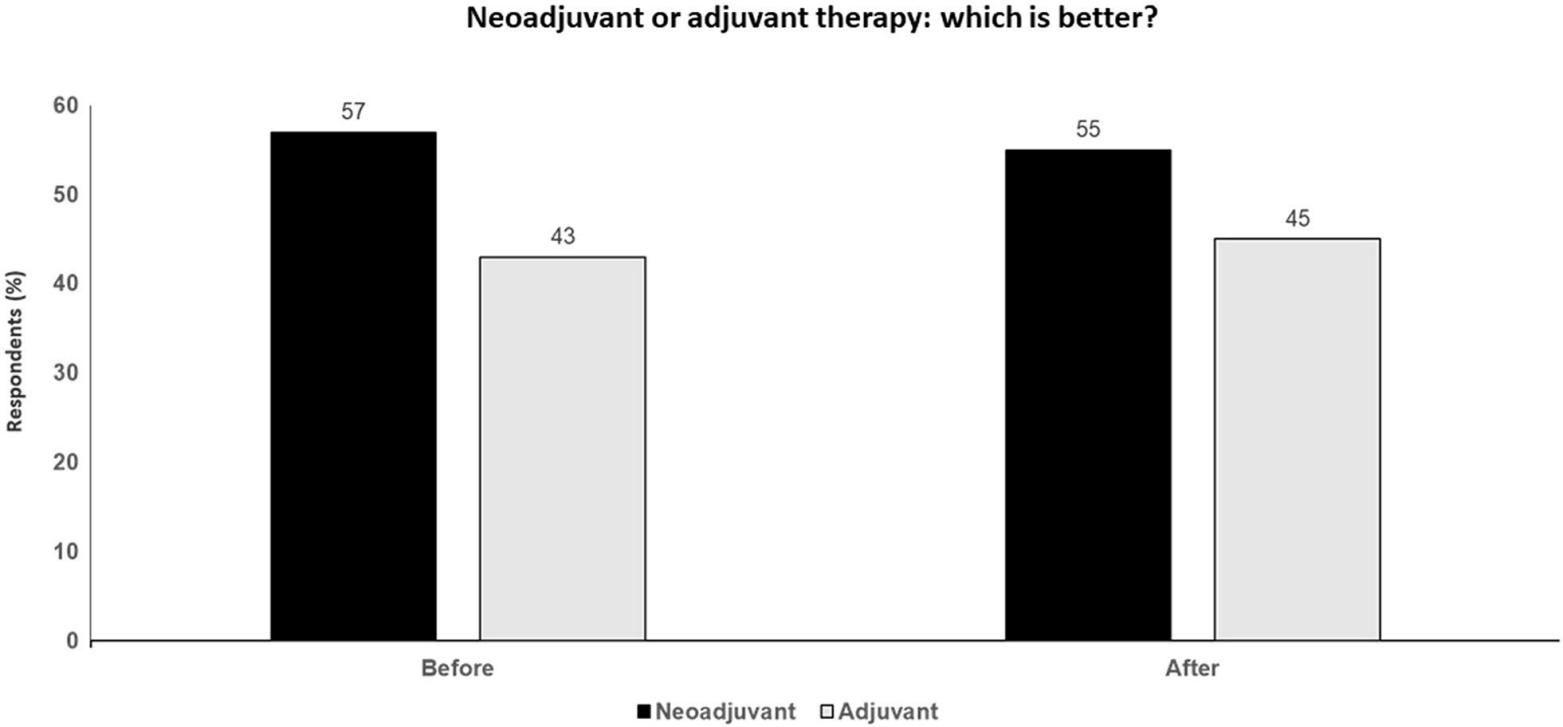
Neoadjuvant or adjuvant therapy; which is better? Audience response before and after debate

Adjuvant therapy is supported by large trials and long-term data, is widely available and the standard of care. In contrast, neoadjuvant therapy is based on a few trials, the small numbers of patients in which do not allow the drawing of meaningful conclusions with regard to clinical effectiveness of the approach. Neoadjuvant expertise is limited and concentrated in a low number of centres and, at this point, is largely an experimental approach that requires rigorous and standardized pathologic and treatment guidelines (Fig. 5).

### Key points

- Neoadjuvant therapy provides the potential for more aggressive treatment, pathological response and toxicity can be evaluated in a short period and can help guide subsequent adjuvant therapy, and can reduce the tumor burden.
- It may result in the activation of many different T cells, with the activation, proliferation, and trafficking of tumor-specific T cell clones already within the tumor microenvironment.
- Neoadjuvant therapy for high-risk melanoma has shown a correlation between pathological response and clinical outcomes, although this requires validation in prospective, randomized studies
- Adjuvant therapy is the standard of care whereas there is an absence of randomized trials and insufficient evidence to make recommendations on the use of neoadjuvant therapy.
- Neoadjuvant treatment is largely an experimental approach that requires rigorous and standardized pathologic and treatment guidelines.

## Conclusions

Counterpoint views from leading experts on five topical issues in melanoma were debated during these sessions. Given the limitations and nature of the format, especially so with the virtual situation necessary this year, each presentation was not intended as a rigorous assessment of the field but rather provided an opportunity to highlight some important areas of debate. It may be that there are no definite answers to these questions, but it is hoped that these discussions can focus attention on these issues, stimulating further debate and encouraging the research needed to improve our understanding of different therapeutic approaches.

## Data Availability

Not applicable.

## Abbreviations

AJCC: American Joint Committee on Cancer
CAR: Chimeric antigen receptor
CLND: Completion lymph node dissection
CTLA-4: Cytotoxic T-lymphocyte-associated antigen
DC: Dendritic cell
DOR: Duration of response
FLT3LG: FMS-related tyrosine kinase 3 ligand
HLA: Human leukocyte antigen
HNSCC: Head and neck squamous cell carcinoma
HR: Hazard ratio
IFN: Interferon
IL: Interleukin
INMC: International Neoadjuvant Melanoma Consortium
KIR: Killer cell immunoglobulin-like receptor
LDH: Lactate dehydrogenase
MDSC: Myeloid-derived suppressor cell
MHC: Major histocompatibility complex
MSLT: Multicenter Selective Lymphadenectomy Trial
MSS: Melanoma-specific survival
NK: Natural killer
NLR: Neutrophil-to-lymphocyte ratio
NSCLC: Non-small-cell lung cancer
ORR: Overall response rate
OS: Overall survival
pCR: Pathological complete response
PD-1: Programmed death-1
PD-L1: Programmed death ligand-1
PFS: Progression-free survival
RFS: Recurrence-free survival
SIRP-α: Signal regulatory protein alpha
SLD: Sum of longest diameters of target lesions
SLNB: Sentinel lymph node biopsy
TAM: Tumor-associated macrophage
TCGA: The Cancer Genome Atlas
TIL: Tumor-infiltrating lymphocyte
Tregs: Regulatory T cells
